# Supplementary figures and images for: Transposon dynamics in the emerging oilseed crop Thlaspi arvense
Source: PLoS Genet. 2024 Jan 31;20(1):e1011141. doi: 10.1371/journal.pgen.1011141 (PMC10881000; doi:10.1371/journal.pgen.1011141)

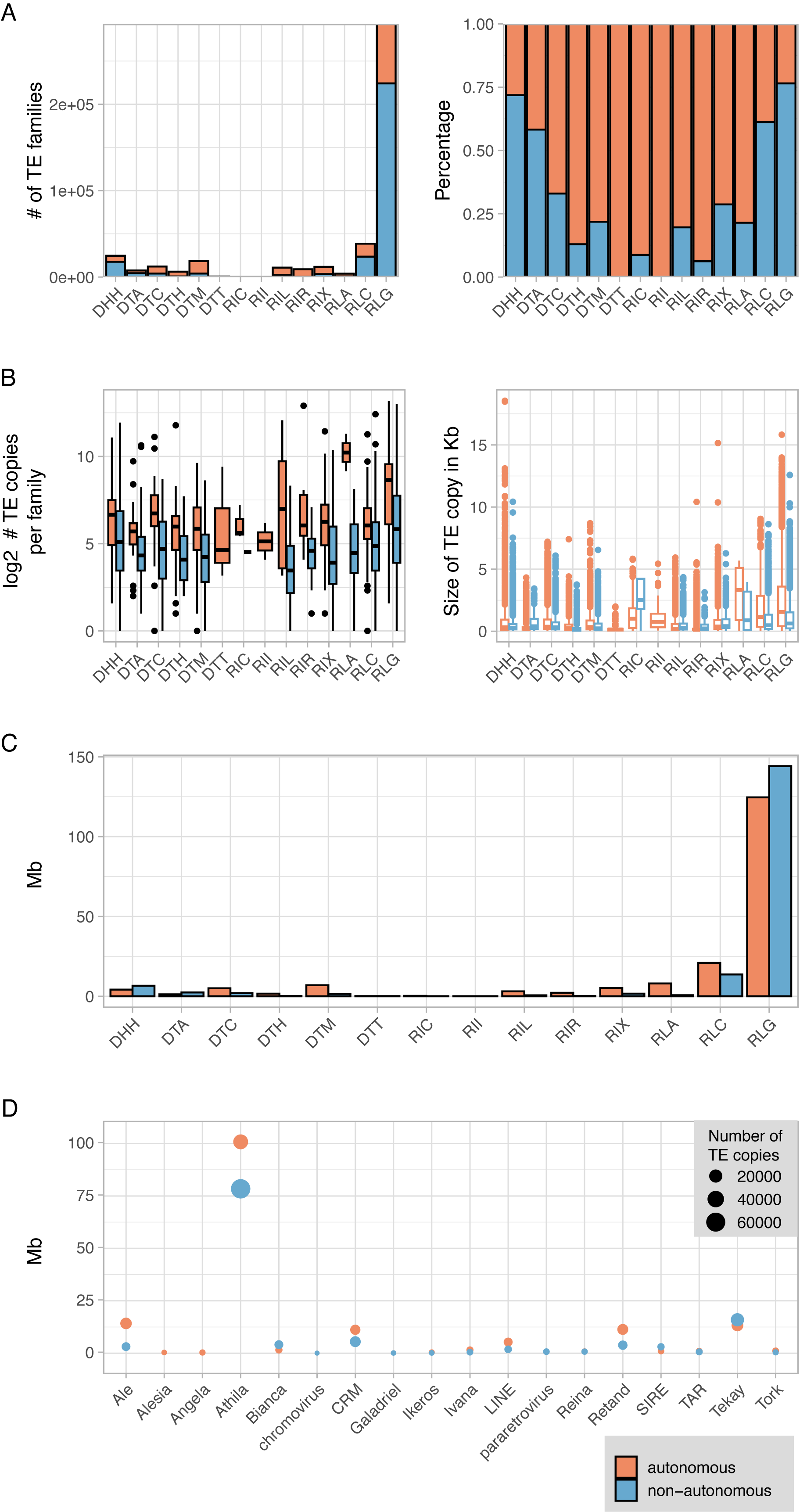

Supplement: S1 Fig — (A) Absolute (left) and relative (right) fraction of autonomous and non-autonomous elements in each TE superfamily. (B) Comparison of the fraction of autonomous and non-autonomous elements in each TE superfamily (left). Size comparison of the TE copies according to their autonomy per superfamily (right). (C) Contribution of each superfamily and their autonomous/non-autonomous fraction to total genome size in Mb. (D) Distribution of size and copy number per LTR retrotransposon lineage. (E) TE expression in autonomous vs. non-autonomous TEs. (TIF) [file pgen.1011141.s004.tif]

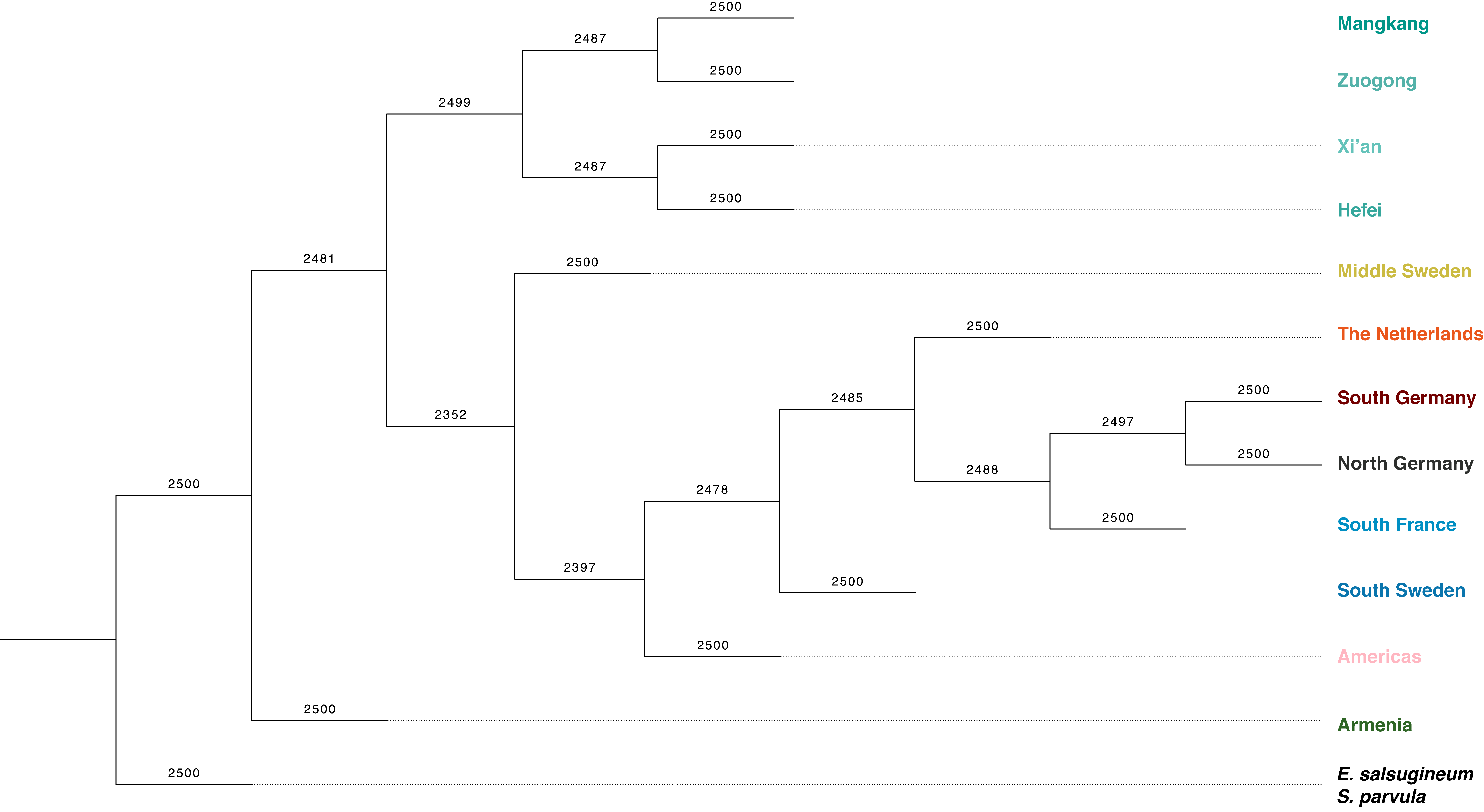

Supplement: S2 Fig — Based on a model without migration, 2,500 bootstraps. Node weights represent bootstrap values. Outgroup species at the bottom. (TIF) [file pgen.1011141.s005.tif]

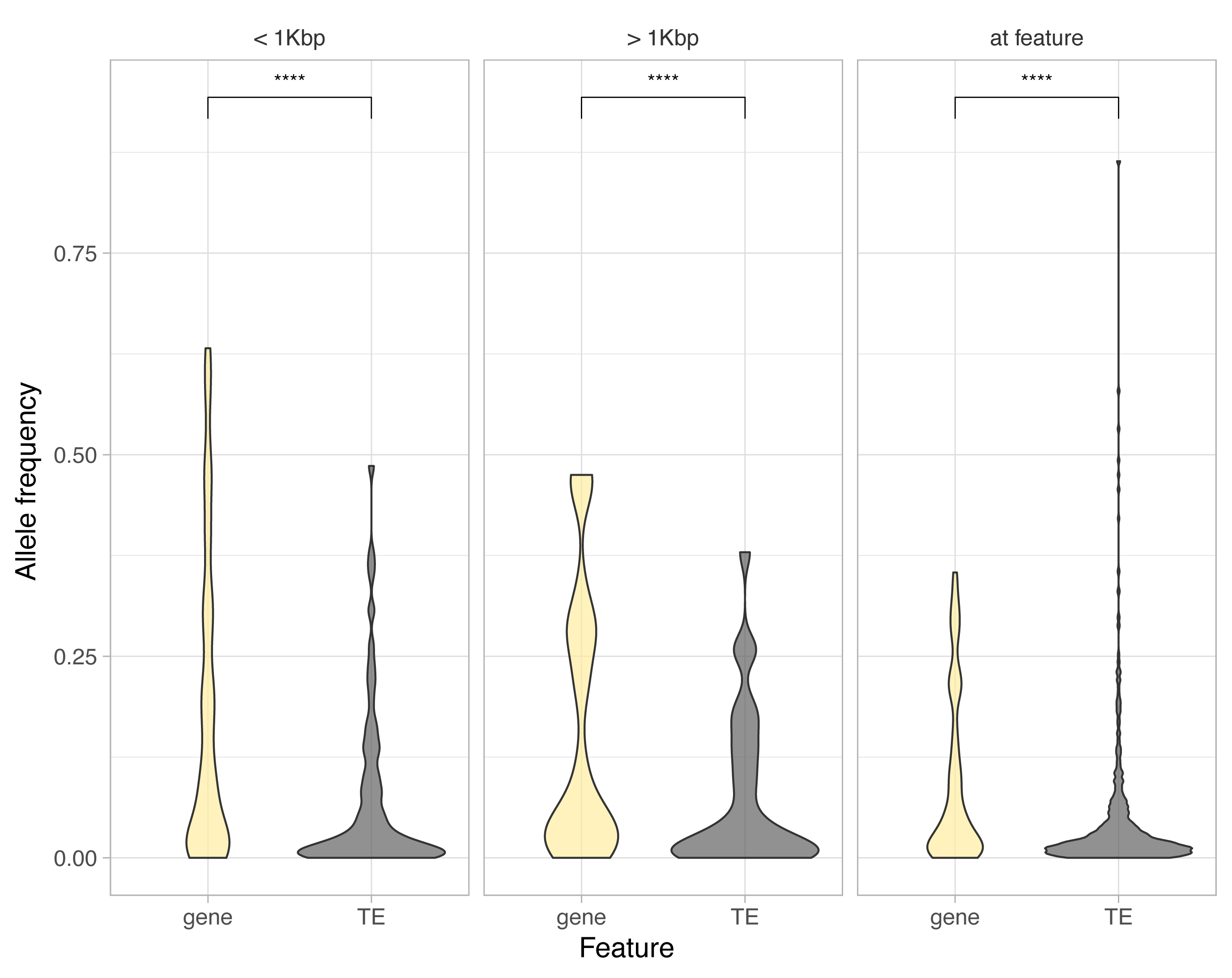

Supplement: S3 Fig — TIP allele frequencies near other TEs are significantly lower than near genes (Wilcoxon Rank Sum test, p < 2.22E-16). (TIF) [file pgen.1011141.s006.tif]

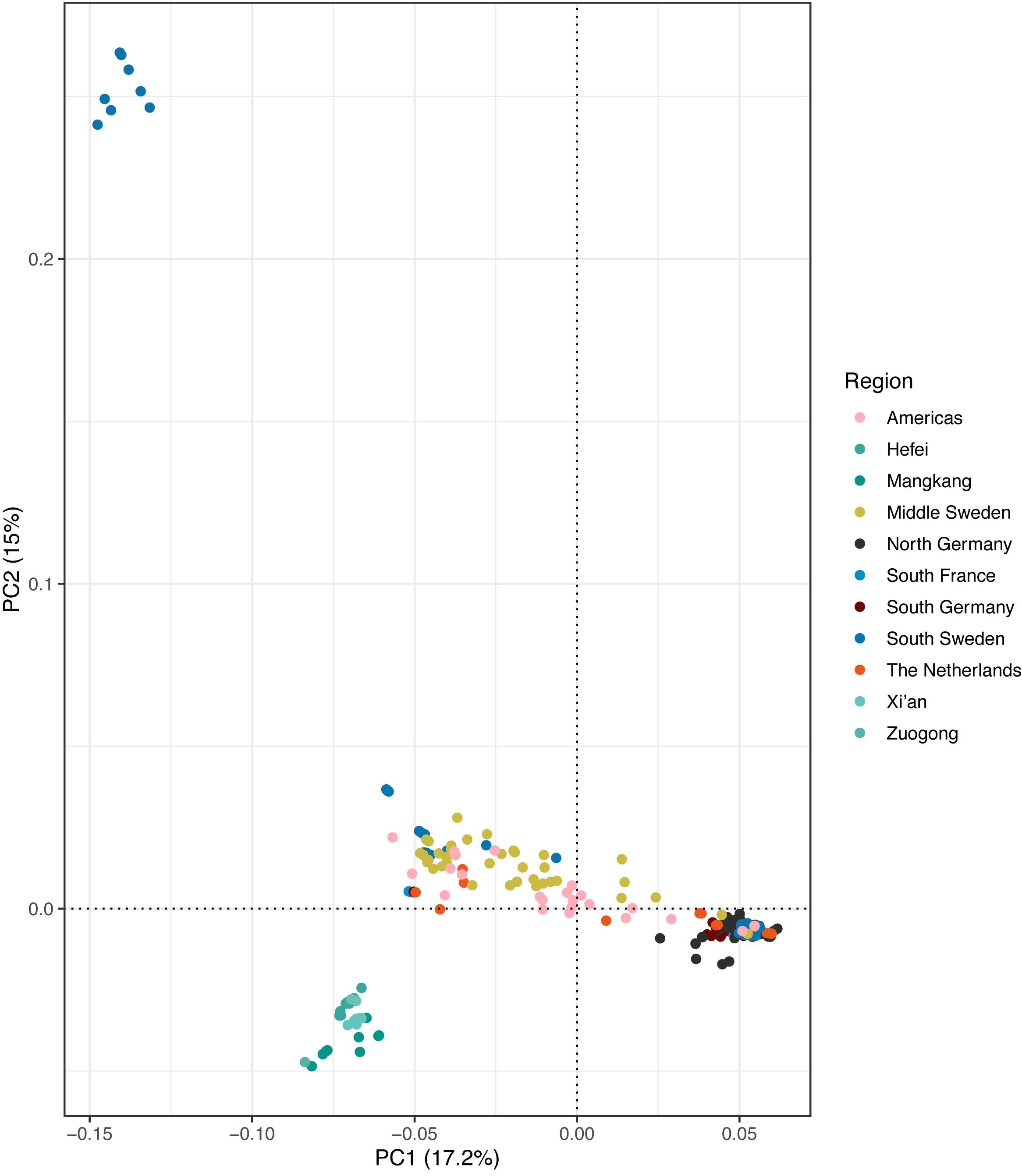

Supplement: S4 Fig — The Armenian accessions, which are outliers in the PCA using all accessions (Fig 2), were excluded from this new PCA analysis, which shows how Chinese and European accessions cluster separately. We also observe part of the south Sweden accessions clustering far from the rest of the European accessions. (TIF) [file pgen.1011141.s007.tif]

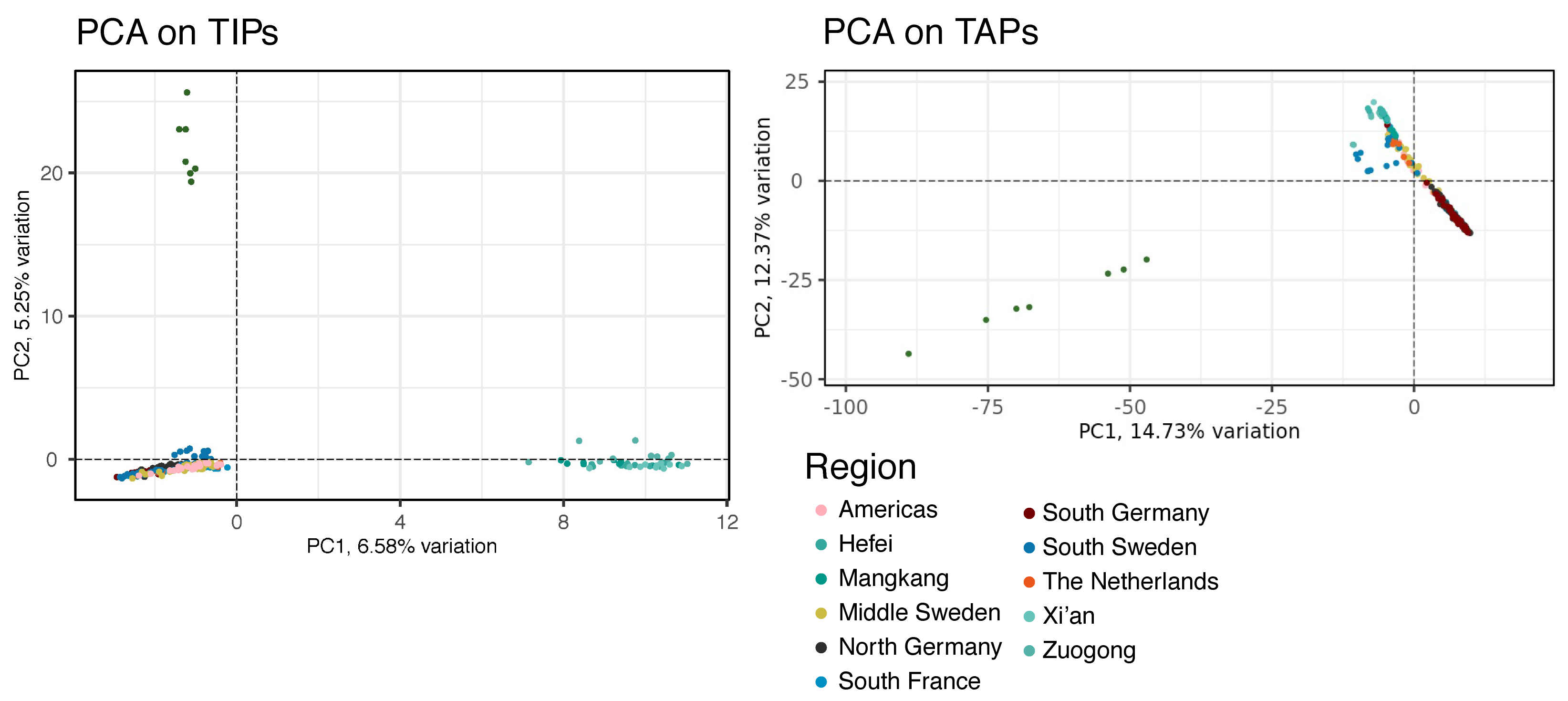

Supplement: S5 Fig — A presence/absence matrix of either TIPs (left) or TAPs, (right) was used as input to calculate PCA. This result recapitulates the clustering pattern observed with the SNP-PCA. (TIF) [file pgen.1011141.s008.tif]

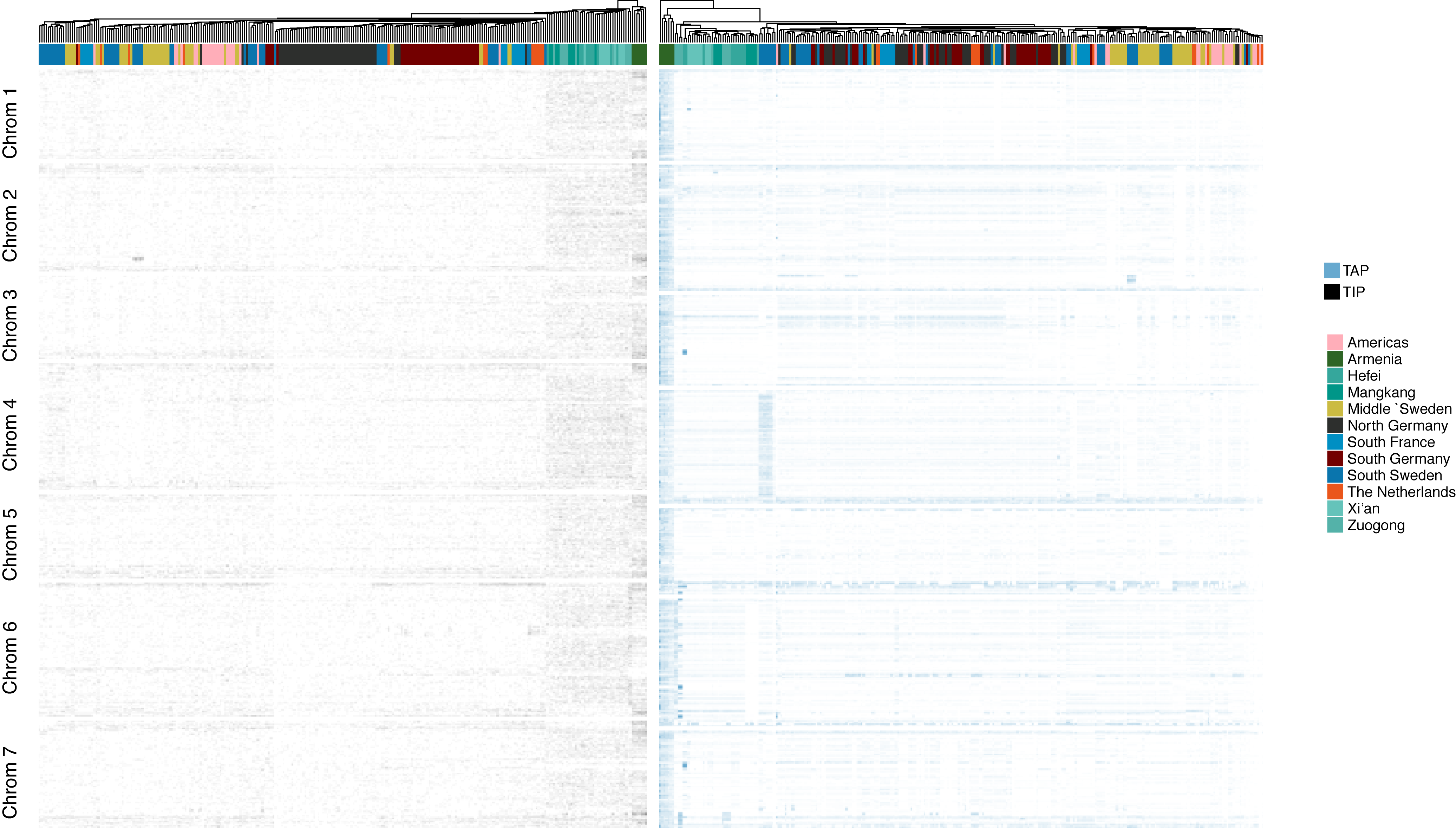

Supplement: S6 Fig — Color columns indicate to which biogeographical population each accession belongs to. (TIF) [file pgen.1011141.s009.tif]

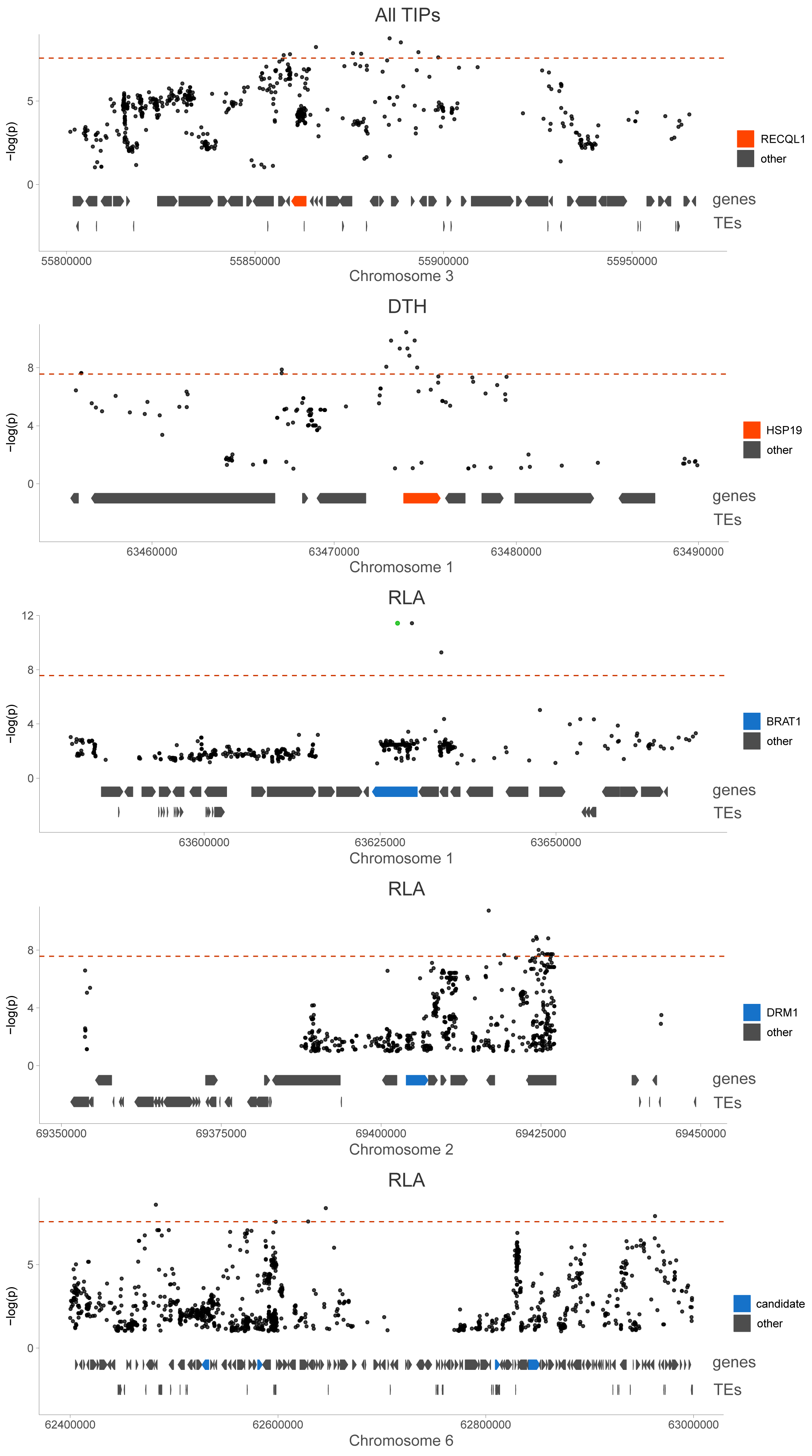

Supplement: S8 Fig — The genome-wide significance (dotted red line) corresponds to a full Bonferroni correction. DNA methylation machinery genes used for the enrichment of a priori candidates are depicted in blue, other genes that might affect transposition in red. The putative knock-out SNP disrupting the function of BRAT1 is depicted in green. (TIF) [file pgen.1011141.s011.tif]

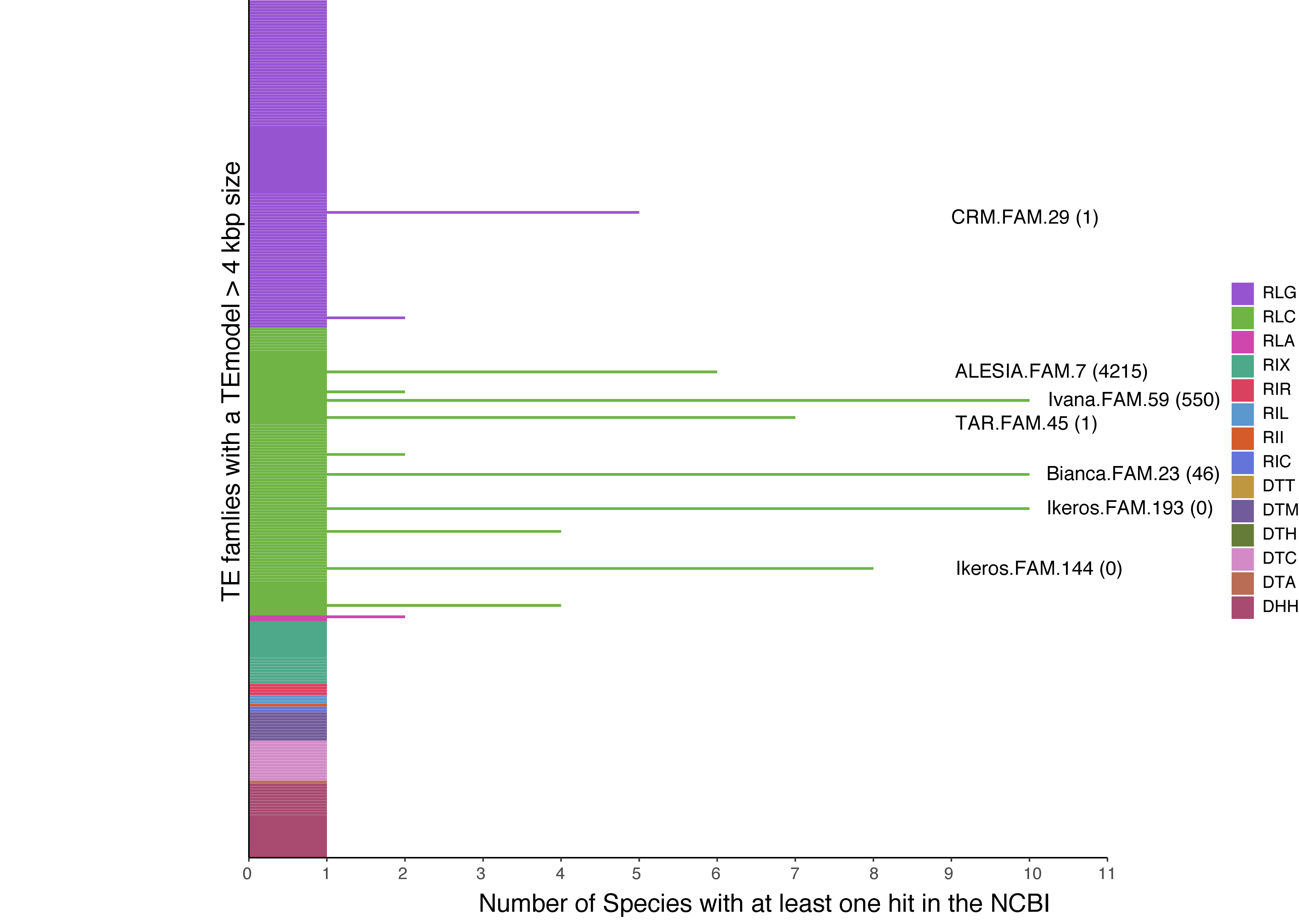

Supplement: S10 Fig — We filtered the matches using the 80/80/80 rule, and further constrained matches to fulfill > 2kb length criteria. The x-axis denotes the number of species with at least 1 hit. Each family has at least one hit, namely T. arvense itself. TE families with more than 5 hits are highlighted. The number of TIPs in T. arvense populations is shown in parentheses for the highlighted families to indicate that there is no obvious correlation between mobility in T. arvense and phylogenetic conservation. (TIF) [file pgen.1011141.s013.tif]
