# Supplementary material for: Transposon dynamics in the emerging oilseed crop Thlaspi arvense
Source: PLoS Genet. 2024 Jan 31;20(1):e1011141. doi: 10.1371/journal.pgen.1011141 (PMC10881000; doi:10.1371/journal.pgen.1011141)
Supplement: Attachment — Submitted filename: Contreras_response_to_reviewers.pdf [file pgen.1011141.s014.pdf]

Response to Reviewers:

Reviewer #1:

Contreras-Garrido et al. present a comprehensive characterization of transposable element (TE) insertion polymorphisms in Thlaspi arvense. Utilizing resequencing data from 280 accessions worldwide, they report extensive variation in the number of polymorphic TE insertions among accessions, with Armenian accessions displaying the highest number of polymorphic insertions. Their exploration of the genetic architecture of this variation through GWAS, using the number of polymorphic TE insertions as a trait, reveals associations with several genes involved in the epigenetic control of TEs. Notably, a recurrent association with a gene encoding a poorly characterized HSP is uncovered. These results are of particular significance, as similar studies in A. thaliana have identified similar epigenetic pathways but through different genes, suggesting species-specific genetic architectures for TE mobilization. Additionally, the identification of a recently expanded Ty1/Copia retrotransposon in Thlaspi arvense, preferentially accumulated within or nearby genes, and containing putative heat-responsive elements analogous to those in the heat-responsive ONSEN retrotransposon from A. thaliana, adds another layer of insight.

Thank you!

The work is sound and meticulously executed, especially given the challenges of identifying TE insertion polymorphisms using short-reads. The identification of recently active TE families absent in the model species A. thaliana, as well as the multiple GWAS findings, are intriguing and pertinent contributions to our comprehension of TE evolution, control mechanisms, and their influence on genome dynamics. Furthermore, the catalog of polymorphisms established here can serve as a valuable resource for researchers and breeders interested in the emerging crop Thlaspi arvense.

1- The observation of a higher load of high-frequency TIPs over gene-rich regions, as noted by the authors on page 8, is unexpected. This conclusion is drawn from Figure 2E; however, this plot may be misleading. Outliers like the few almost fixed TE insertions near chromosome ends can create the impression of an overall trend. It may be more informative to display the average allele frequency by genomic windows (e.g. 100/500Kb windows) instead.

We agree that windowed averages are preferable. We have now averaged the TIP allele frequency over 100 Kb windows across the genome and updated panel Figure 2E:

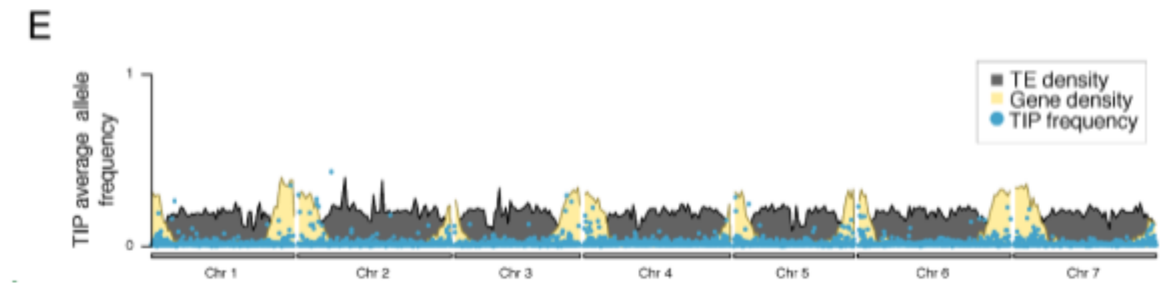

To further support our originally intended assessment of TIPs over gene-rich regions, we also provide Figure S3, where we quantify the differences in TIP allele frequencies for TIPs near or over genes versus TIPs near or in TEs. We differentiate between TIPs that directly overlap features (genes or TEs), that are within 1 kb from the nearest feature, or that are over 1 kb apart from the nearest feature. This analysis confirms that TIP allele frequencies

near TEs are lower than near genes, supporting the primary observation that “What speaks against this view is that TIPs in the gene-rich fraction of the genome, near the telomeres, have higher allele frequencies (Fig. 2E), while TIPs in the pericentromeric regions are more abundant, but have lower allele frequencies (see Fig. S3 for a statistical assessment).”

Furthermore, the challenge in detecting polymorphic insertions within TE-rich regions compared to unique sequences could skew the allele frequency spectrum (AFS) towards lower values in the former. One possibility would be determining the mappability of Illumina short-reads at each insertion site to distinguish between “true absences” of TE insertion polymorphisms from a “lack of informative” reads. The latter should be considered as "NA" rather than "absences."
This is an excellent suggestion and we agree that consideration of mappability is of central importance for studies of TE insertion polymorphisms. We took this into account by masking regions of the genome with aberrant read coverage, which we now explain more fully in the methods: “Next, we mapped short reads from the reference accession MN106 to the reference genome to identify regions of aberrant coverage, using sample SAMEA9464759 from ENA project PRJEB46635 [26]. We calculated read coverage (RC) in 100 bp windows, adjusted for GC content [70], and excluded windows with abnormal coverage, arbitrarily defined as threefold lower or higher than the genome wide, GC content adjusted mean. Any TIPs in these regions, which corresponded to ~16% of the reference genome, were excluded from the final dataset.”

2- GWAS were conducted on TIPs, which are defined as non-reference insertions in this work. Since the number of TIPs is expected to correlate with the genetic distance to the reference genome, as suggested in Figure 3B, the GWAS might primarily capture this reference bias. To mitigate such bias, it might be desirable to employ the number of rare TIPs, as well as common TAPs, per accession as a proxy for TE mobilization. These numbers are expected to represent the most recent non-reference and reference insertions, respectively.
We agree on the importance of this point. Regions of a genome that diverged earlier than other regions of a genome from the reference had more time to accumulate TIPs. We estimated the age of each TIP based on the maximum pairwise divergence (number of SNPs) between the genomes in the regions flanking the insertion (Baduel et al. 2021). With this, we found a higher proportion of older TIPs in Armenian accessions, which have genomes that diverged on average a much longer time ago than other accessions:

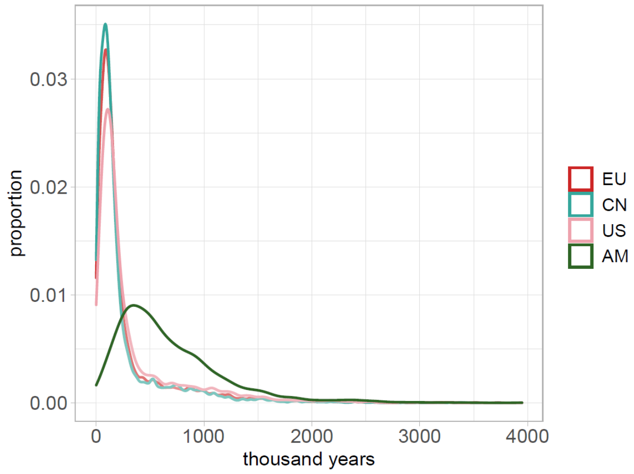

To mitigate reference bias, we also ran GWAS using only TIPs estimated to be younger than 500,000 years (about 88% of all TIPs). Except for R2 retrotransposons (RIR), results were largely unchanged. In the case of RIR, p-value distributions became very skewed, because too many accessions had no recent TIPs. We therefore opted to keep all TIPs for GWA analysis, but to be transparent, we added the following to the manuscript:

1. TIP age estimates: We report these in the main text, we added a description to the methods, we added the estimated ages of all TIPs to our Zenodo repository, https://doi.org/10.5281/zenodo.10161730, and we added the code to our GitHub, https://github.com/acontrerasg/Tarvense_transposon_dynamics/tree/main/TIPs_age.
2. We report the GWAS results with TIPs younger than 500,000 years in the main text and in an additional supplementary figure (Figure S9).

Regarding common TAPs, we did not include the reference genome in the GWAS, because non-reference and reference insertions are determined with very different methods, which almost certainly will introduce a serious bias. Previous studies in *A. thaliana* (e.g., Baduel et al., 2021) have taken a similar approach.

Minor comments:
Page 3. “In the Ty3 superfamily, Ale elements…”. Ale does not belong to Ty3 superfamily.
Corrected.

Page 14. “Although studies in A. thaliana have highlighted differences…., they are not as striking compared to the evidence we found here”. I am not sure I follow the reasoning behind why the findings presented here are considered more striking. Please provide further elaboration on this argument.
Our sentence was only pertaining to the difference between Class I and Class II TE families, but we realized that our phrasing was misleading. The reader should determine how significant the differences between *A. thaliana* and *T. arvense* are, and we have therefore removed “not as striking”, and rephrased this as:
“Although studies *in A. thaliana* have highlighted differences in the genetic control of methylation and mobility of the two classes of transposons, GWA for CHH methylation of TE families did not produce very different signals for class I and II families. The same was true for TIP-counts of different families and superfamilies as phenotypes.”

Page 14. “…differences in the genetic control of silencing of class I and class II TEs is further supported by … DNA methylation spreads from class I TE insertions, but not from class II TE insertions” . It is unclear from this work how methylation spreading relates to the differential control of TEs. Please place these results in the context of current knowledge regarding DNA methylation spreading and the various epigenetic pathways involved.
We apologize for this leap in argumentation, which on second thought is not justified. In *A. thaliana*, spreading of methylation from some – but not all – siRNA producing loci is well established (Henderson and Jacobsen 2008; Daxinger et al. 2009; Ahmed et al. 2011), and it has been linked to the pathway that silences the siRNA producing loci, RdDM, but not to the characteristics of a given TE superfamily. We hypothesized that the difference between Class I and Class II in the methylation spread could be due to the trans-acting machinery, but we can of course not exclude that it is due to special cis-sequence differences.
We therefore changed our discussion from: “Our interpretation that natural genetic variation in *T. arvense* points to differences in the genetic control of silencing of class I and class II

TEs is further supported by methylome evidence, where we found that DNA methylation spreads from class I TE insertions, but not from class II TE insertions."
To:
"That class I and class II TEs in *T. arvense* apparently differ in their genetic requirements for silencing can be potentially linked to our observation that DNA methylation spreads more rapidly from class I than class II TE insertions in this species."

Page 14. "Modulation of gene expression via intronic insertions". It is unclear how intronic insertions can modulate gene expression. Please expand on this point and incorporate relevant literature.
Response: We have tried to make this clearer:
"TE insertions in exons might disrupt genes, while intronic insertions might modulate alternative splicing or reduce the accumulation of correctly spliced transcripts. An example is provided by *A. thaliana* accessions in which an intronic COPIA insertion in the disease resistance gene *RPP7* shifts the balance between full-length and truncated transcripts [54] (Tsuchiya and Eulgem 2013)."

Reviewer #2:
The manuscript by Contreras-Garrido & Galanti and coauthors explores the transposable element landscape in natural populations of Thlaspi arvense across different geographic regions. The authors classified the TEs and their genetic variability within the samples/populations and explored the meaning of the variability. The findings are intriguing and contribute to the knowledge of the emerging oilseed crop Thlaspi arvense. Although the authors used GWA to analyze TE insertion polymorphisms in 280 wild accessions, other datasets would improve the manuscript, allowing important conclusions to be drawn.
Thank you! As suggested, we added a new set of expression data (Nunn et al. 2022). This new dataset provides further evidence in support of the notion that in *T. arvense* autonomous TEs are overall more highly expressed than their non-autonomous counterparts (Figure S1).

1. Pg 3 - …ongoing mobilization activity (13,18,56). Is unclear what those numbers mean. Could the authors clarify?
Thank you for pointing this out. To avoid confusion, we changed the formatting style of the references in the manuscript to square brackets, so references are more easily distinguished from other numbers.

2. Fig 1B – A color key in the panel (not only in the legend) will help the reader understand the circular plot better/faster.
Thanks. Color legend added to Figure 1B and color schemes in both Figure 1A and 1B made more consistent.
>> Updated panel 1B with legend:

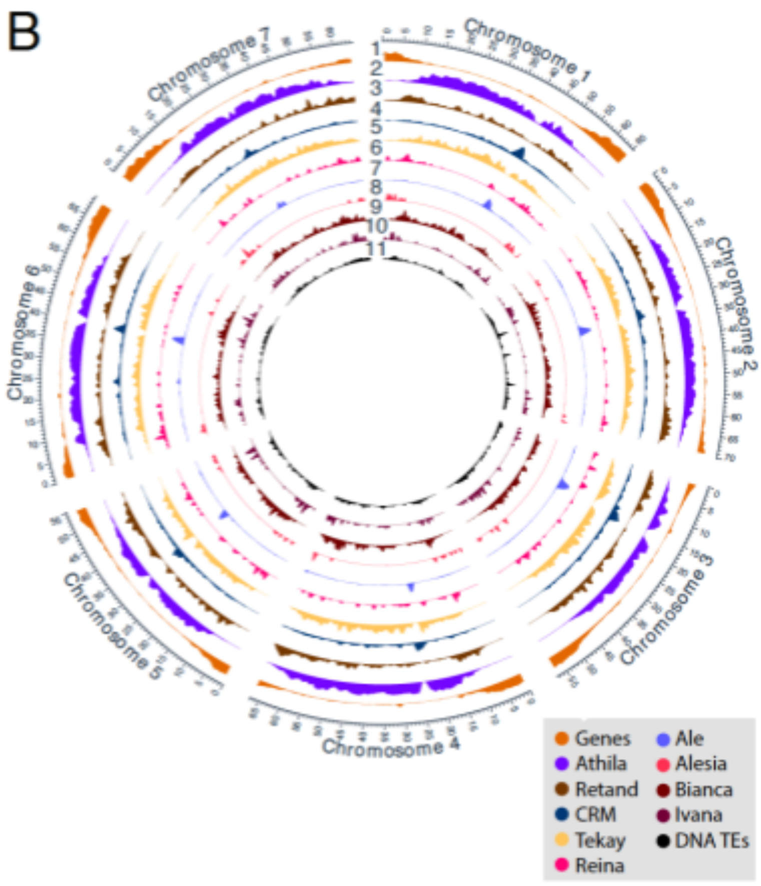

3. Transcriptome (or other expression analysis) would help to address if the autonomous TEs are active or silenced.

Excellent suggestion. To do so, we used the RNA-seq dataset from (Nunn et al. 2022). TE expression was quantified using TEspeX (RPM: raw counts / total mapped reads x 1M). We used a non-parametric Wilcoxon rank-sum test to compare expression between autonomous and non-autonomous TE families.

>> New panel added for Figure S1E:

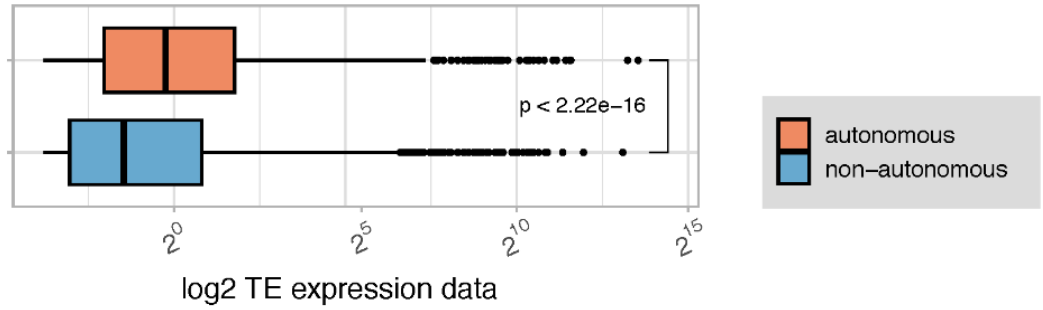

Autonomous TE families have indeed higher levels of expression. This may, however, not necessarily entail higher activity, as more mRNA can also mean more efficient small RNA mediated targeting and thus silencing.

4. Does the methylation analysis not include TEs-body, only the surrounding regions? Are the TEs bodies methylated? Which tissue was used for the WGBS?
The methylation analysis used the dataset of (Galanti et al. 2022) obtained from leaf tissue, specifically the third or fourth true leaf. Because the data are from wild-collected accessions, we could not map these reads to the non-reference TE insertions (TIPs) themselves, since by definition these TIPs are missing from the reference genome. We therefore focused on the flanking regions of the insertions, which are (usually) present in the reference.

5. Is tempting and makes sense that RdDM would be responsible for the methylation, however, more information is necessary to conclude that. Do the TEs generate mRNA and sRNAs? Other silencing mechanisms are possible, for instance, alternative TEs silencing mechanisms such as in Habu et al., 2006 and Rangwala & Richards, 2007.
As the reviewer points out, TE silencing is the combined result of several interacting pathways. Because we observe changes in methylation for CHG and CHH sites, which do not depend on MET1, and because the spread of methylation in *A. thaliana* is RdDM mediated (Ahmed et al. 2011), we focused our discussion on RdDM. Also of relevance is that recent work has shown that MOM1, initially identified because it can mediate silencing independently of changes in methylation, also relies on the RdDM machinery (Li et al. 2023).

6. The common name (“penny cress”) should be used in the abstract
Done.

Reviewer #3
Transposable elements (TEs) account for a large proportion of eukaryotic genomes and contribute to evolution of traits through generating new genic or regulatory sequences. However, our understanding on how and to what extent transposable elements may contribute to trait evolution in a given species are limited. In the current manuscript, the authors analyzed 280 wild accessions of an emerging oil crop, Thlaspi arvense, to obtain a map of TE variations in this population. Thlaspi arvense is a member of the Brassicaceae family and its genome contains a large proportion of TE compared to most of other Brassicaceae species with genome sequences available. They performed a comprehensive analysis on TE diversity in Thlaspi arvense genome and in the wild population. The new findings are a new Ty1/Alesia lineage that closely related to protein-coding genes and new host factors associated with TE activity. The manuscript is well organized and well written. I enjoyed reading the manuscript. I summarized the strength and weakness of the manuscript that may help the authors to improve the manuscript.4
Strength:
TE activity might be very difference from species to species. Therefore, a new model for studying TE activity and TE regulation is always attractive. The authors performed a comprehensive analysis of TE polymorphism and identified host factors associated with TE regulation in 280 wild accessions of the emerging old crop, Thlaspi arvense. Overall, the results suggest that TE regulation in Thlaspi arvense is different from Arabidopsis thaliana. This warrants further studies to explore these host factors and impact of TEs on host adaption.
Thank you.

Weakness:
The authors reported several interesting observations. However, the analysis could be extended to provide detailed evidence to support the conclusion. For example, the authors identified several candidate genes associated with TE activity. In Figure S8, the authors showed GWAS signals, which suggest that multiple genes were included in the peak region. The authors could provide detailed analysis on how they determine the candidate genes using haplotype or gene expression analysis.

Many thanks for this valuable suggestion. We indeed found that at least one of the SNPs is highly likely to be causal: "Some of our GWA peaks extend over several genes and might reflect associations with less well characterized genes, but others have the strongest associations in individual genes such as *HSP19* and *BRAT1* (Fig. S8). For *HSP19*, the top SNPs are located in introns and it is difficult to predict their effect. *BRAT1* has two highly significant, fully linked SNPs in exons 1 and 4. The SNP in exon 4 (Chr1:63627484) introduces a stop codon that removes part of the ATPase domain and the entire chromatin binding bromodomain, and this mutation almost certainly completely eliminates BRAT1's anti-silencing activity [7]."

Regarding the "candidates" in the last panel of Figure S8, those are the "a-priori candidate" genes used for enrichment analysis of DNA methylation machinery genes. These are orthologs of *A. thaliana* genes with proven roles in DNA methylation (Galanti et al. 2022) and such enrichment analysis has been used extensively to quantify how much GWAS results overlap with a-priori expectations (e.g., (Sasaki et al. 2019; Sasaki et al. 2022; Atwell et al. 2010; Kawakatsu et al. 2016)). We compared how much the mobility of specific TE superfamilies might be associated with DNA methylation genes and genes that could affect transposition in alternative ways such as *HSP19* or *RECQL1*. We clarified this by adding a sentence to the results section and referring to *"a-priori"* candidates in the captions of Figure 4 and Figure S8. We modified Figure S8. and highlighted DNA methylation *a priori* candidates in blue, as in Figure 4, to make this clearer.

Similarly, the authors claimed heat responsive elements in Alesia.FAM.7. Instead of stating that "It is conceivable that this heat-responsive, euchromatophilic Alesia family rewires gene regulatory networks between and within Brassicaceae species." The authors could setup an experiment to prove Alesia.FAM.7 transcription can be induced upon heat stress.

We agree it would be good to have additional experimental evidence for the environment-dependent activity of these elements. Unfortunately, because heat stress has not been studied much in *T. arvense* so far, this would require an extensive investigation of what constitutes heat stress in this species. We will keep this in mind, however, for future experiments, to determine experimentally TE mobilization.

Minor concerns:
1, In page 3, the authors classified TEs into autonomous TEs and nonautonomous TEs but I did not find the related description in the method section. Please add these details in the method.

We now include a more detailed description and definition of classification of autonomous or non-autonomous elements:

“Using these identified protein domains, we evaluated whether a given TE family is autonomous, i.e., whether it codes for the entire machinery needed for transposition. An LTR retrotransposon family was considered autonomous with the following domains identified by DANTE: retrotranscriptase, RT; capsid related domain, GAG; RNase H, RH; protease, PROT; and integrase, INT. Autonomous non-LTR retrotransposons, LINEs, had to contain: retrotranscriptase, RT. DNA TE families had to contain: transposase, TPase. DNA TEs of the Helitron superfamily had to contain in addition: DNA helicase, HEL.”

2, In page 5, the authors stated that “Because a substantial part of the reference genome consistent of autonomous, like still active, TE families, we want to learn how variable TE content is in a large collection of natural accessions”. First, autonomous TEs do not mean it is active. This is not accurate. The author can safely remove “like still active” in this sentence. Second, the authors may want to say something after “learn how variable TE content is”. Please finish the sentence.
We have removed the “likely still active” part: “To learn how TE mobility has shaped genome variation at the species level, we surveyed differences in TE content in a large collection of natural accessions.”

3, In page 9, the authors claimed that “In all three epigenetic contexts (CG, CHG, CHH), we found a significant increase of methylation up to 1 kb around class I, but not around class II TE insertions”. Although the spreading of DNA methylation by class I TE has been reported, further analysis could be performed to provide some case examples of methylation spreading caused by these new TE insertions.
We agree that this is an interesting avenue to explore. Unfortunately, we do not have RNA-seq data for the analyzed populations. We hope that our indirect evidence will encourage studies to collect such data.

Have all data underlying the figures and results presented in the manuscript been provided?

Large-scale datasets should be made available via a public repository as described in the *PLOS Genetics* data availability policy, and numerical data that underlies graphs or summary statistics should be provided in spreadsheet form as supporting information.
Reviewer #1: Yes
Reviewer #2: Yes
Reviewer #3: No:
Thank you for noticing this. The record should have been available at ENA under project ID PRJEB62093, but apparently there were issues with ENA uploading the files. We have re-uploaded all short-read data and they are now available. There is also a data set of long reads that has been uploaded and that is currently being processed; it should be available in the next few days.
We include all the data generated in this work in the Zenodo repository at https://doi.org/10.5281/zenodo.10161730
